# Relationship between short and long-term glycemic variability and oxidative stress in type 1 diabetes mellitus under daily life insulin treatment

**DOI:** 10.20945/2359-3997000000338

**Published:** 2021-03-19

**Authors:** Tatiana Valente, Fernando Valente, Maria Beatriz Bastos Lucchesi, Giovana Rita Punaro, Margaret Gori Mouro, Monica Andrade Lima Gabbay, Elisa Mieko Suemitsu Higa, Sergio Atala Dib

**Affiliations:** 1 Universidade Federal de São Paulo Centro de Diabetes Divisão de Endocrinologia São Paulo SP Brasil Departamento de Medicina, Divisão de Endocrinologia, Centro de Diabetes da Universidade Federal de São Paulo, São Paulo, SP, Brasil.; 2 Universidade Federal de São Paulo Divisão de Nefrologia Departamento de Medicina São Paulo SP Brasil Departamento de Medicina, Divisão de Nefrologia, Universidade Federal de São Paulo, São Paulo, SP, Brasil.

**Keywords:** Glycemic variability, glycemic fluctuations, oxidative stress, type 1 diabetes

## Abstract

**Objectives:**

The purpose of this study was to investigate the heterogeneity of the association between glycemic variability and oxidative stress markers in T1DM patients under daily life insulin treatment.

**Subjects and methods:**

We studied, in a cross-sectional analysis, 76 T1DM patients without clinical chronic diabetes complications and 22 healthy individuals. Were evaluated the short-term glycemic variability (STGV), long-term glycemic variability (LTGV), oxidative stress markers [8-isoprostaglandin-F2α (Ur-8-iso-PGF2α), nitric oxide (NO), thiobarbituric acid reactive substances (TBARS) and erythrocytes reduced/oxidized glutathione (GSH/GSSG)] and biochemical dosages (glycaemia, HbA1c, lipidogram, albuminuria).

**Results:**

Plasmatic NO was positively associated with LTGV (last year average of HbA1c) (8.7 ± 1.6% or 71 ± 18 mmol) (rS: 0.278; p: 0.042). Plasmatic TBARS, erythrocytes GSH/GSSH and Ur-8-iso-PGF-2α didn't show correlation with glycemic variability. GSH/GSSG was inversely correlated with LDL-cholesterol (rS: - 0.417; p: 0.047) and triglycerides (rS: −0.521; p: 0.013). Albuminuria was positive correlated with age (rS: 0.340; p: 0.002), plasmatic NO (rS: 0.267; p 0.049) and TBARS (rS: 0.327; p: 0.015).

**Conclusion:**

In daily life insulin treatment, young T1DM patients have higher plasmatic NO than healthy subjects. However, the correlation between glycemic variability and oxidative stress markers is heterogeneous. Lipid profile and albuminuria are associated with different oxidative stress markers. These data collaborate to explain the controversial results in this issue.

## INTRODUCTION

Large randomized studies have established that early and persistent glycemic control during diabetes mellitus natural history, reduces the risk to develop both micro and macrovascular chronic complications of this disease (1). The initial and prolonged effect of overall glycemic control in this process is part of the “metabolic memory”. This concept supports the adoption of a precocious, aggressive and continuous treatment approach, at least, since the clinical diabetes diagnosis (2).

It is known that persistent hyperglycemia results in overproduction of oxygen free radicals, which contributes to the beginning and progression of chronic diabetes complications (3). However, recent evidence suggests that acute glucose fluctuations may accelerate the development of diabetes chronic complications (4) more than chronic hyperglycemia (5), by triggering oxidative stress (6). Hyperglycemic spikes sometimes seems to be high enough to activate oxidative stress that persists during subsequent periods of normoglycemia, but too brief to affect the HbA1c (7). So far, there is no “gold standard” parameters for determining glucose variability (3). The Mean Amplitude Glucose Excursions (MAGE) is the most used for Continuous Glucose Monitoring System (CGMS), and standard deviation (SD) and/or coefficient of variation (CV) for self-monitoring blood glucose (SMBG) curves (3). Majority of studies are based on the 7-point glucose profiles, that may not capture the full degree of variability that is observed in the CGMS (8).

There is a paucity of studies on the effects of glucose fluctuations on routine clinical management and oxidative stress in patients with type 1 diabetes mellitus (T1DM) (9).

Many markers have been used to evaluate oxidative stress degree in patients with diabetes such as urinary 8-iso-prostaglandin-F2α (8-iso-PGF2α), glutathione, barbituric acid reactive substances (TBARS) and nitric oxide (NO).

8-iso-PGF-2α is a specific isoprostane formed from free radical oxidation of arachidonic acid and is an excellent reflection of activating oxidative stress throughout the body (10).

Glutathione is one of the key antioxidants involved in protecting cells from damage by reactive oxygen species. It exists in the body in its reduced (GSH) and oxidized form (GSSG), acting directly or indirectly in many important biological processes including protein synthesis, metabolism and cell protection (11). From the ratio of reduced glutathione/oxidized glutathione (GSH/GSSG) it is possible to do an analysis of the antioxidant defense system.

TBARS are reactive thiobarbituric acid substances that, in vitro, have proved as potent oxidative stress parameter. However, it is not a very sensitive method to evaluate oxidative stress (12).

Nitric oxide (NO) plays an important role in modulating endothelial function, with several antiatherogenic actions, however depending on the environment, it can be potentially toxic. NO is generated from oxide nitric synthase (NOS) of which there are three forms: two constitutive types [brain (bNOS) and endothelial (eNOS)] and one inducible type (iNOS). Glycemic fluctuations reduce eNOS activity and increase iNOS expression, leading to an overproduction of NO. Various impairments in NO pathways have been reported in T1DM both in animal models and humans (13). However, in vivo, it is not clear whether the defect is in basal or stimulated NO synthesis, NO bioavailability, responsiveness to NO, or perhaps all of these.

The aim of this study was to investigate the heterogeneity of the association between short and long term glycemic variability with markers of oxidative stress in patients with type 1 diabetes mellitus in real word.

## SUBJECTS AND METHODS

### Study design

Study protocol was approved by the local ethics committee of Federal University of São Paulo (CAAE registration number generated in Plataforma Brasil was 02703212.6.0000.5505) and participants or their legal guardians gave written informed consent.

It was a prospective study, conducted from 2013 to 2014 at Diabetes Center of São Paulo Federal University and enrolled 76 T1DM and 22 healthy controls individuals.

Inclusion criteria were T1DM patients aged between 12 and 45 years old, diagnosed for at least five years, without clinical chronic diabetes complications, and had at least two HbA1c in the last year from the same laboratory. Exclusion criteria were reported patients with subcutaneous continuous insulin infusion (CSII), infectious disease, during menstrual flush, pregnancy, smoking, and any inflammatory process in activity.

One hundred and thirty medical records were reviewed to select patients for the study. Fifty-four patients were ineligible because they presented some of the exclusion criteria. Seventy-six T1DM filled the criteria inclusion, were contacted by phone or during a routine clinical appointment and participated in the study. Twenty-one patients were excluded from some analysis because they didn't follow the protocol, didn't collect blood sample or showed during randomization, any inflammatory or infectious disease. However, these 21 patients presented demographic characteristics like the other participants included.

Twenty-two controls without any chronic disease or other exclusion criteria, were enrolled for the study, and were adjusted for age and body mass index (BMI).

### Study protocol

At subject study entry, a subcutaneous continuous glucose monitoring system (CGMS) sensor (Medtronic MiniMed^®^, Northridge CA, USA) was inserted subcutaneously into the abdominal region and calibrated.

Fingertip capillary blood glucose was measured at least three times per day and values were used to titrate CGM meters. The monitor was removed after three days, and data was downloaded and analyzed using CGMS Software version 3.0 (CA, USA). Eight to twelve hours fasting venous blood samples were collected on the Day 1 and 8 hours overnight urinary samples on Day 3 of this continuous interstitial glucose monitoring period. Collected blood samples were stored at −80°C until laboratory testing for analysis of oxidative stress biomarkers and other laboratory tests.

### Glycemic variability

Short-term glycemic variability (STGV) was defined by standard deviation (SD) of glucose values during continuous subcutaneous glucose monitoring system (CGMS) over three consecutive days (between days). Long-term glycemic variability (LTGV) was accessed by mean glycated hemoglobin (HbA1c) of last year (visit to visit) (14). These measures were obtained in both T1DM patients and healthy controls.

### Oxidative stress biomarkers

#### 8-iso-prostaglandin-F2α

8-iso-PGF2α was measured by an enzyme-linked immunosorbent assay (ELISA) (EIA-catalog number ADI-I-900-010) (ALPCO-US) from individuals' eight hours overnight urinary samples. Urine was diluted fourfold with sample dilution buffer containing a final concentration of ~0.1 mM TPP (triphenylphosphine, 0.03-0.05 mg/mL). TPP is an antioxidant which looks like a precipitate in samples as it does not easily dissolve. Before using stored samples containing TPP, spin samples at 10,000 rpm for 5 minutes to separate the precipitated TPP and other particulates from sample solution. The inter-assay coefficient of variation was 11.3% and range between 600-1400 pg/mL.

#### Nitric oxide (NO)

Plasmatic NO was quantified using chemiluminescence method; Model 280 Nitric Oxide Analyzer (NOA^TM^), Sievers Instruments, Inc. (Boulder, CO, USA), a high-sensitivity detector for measuring nitric oxide, based on gas-phase chemiluminescent reaction oxide between nitric oxide and ozone, as described elsewhere.


$$\begin{array}{l} {\text{NO}}^{-}+{\text{O}}_{3}\to {\text{NO}}_{2}{}^{-}+{\text{O}}_{2}\\ {\text{NO}}_{2}{}^{-}\to {\text{NO}}_{2}{}^{-}+\text{hV} \end{array}$$

Emission of a photon from electrically excited nitrogen dioxide is in the red, near-infrared region of the spectrum and is detected by a thermoelectrically cooled red-sensitive photomultiplier tube. Sensitivity for measurement of NO and its reaction products in liquid samples is ≈1 pmol.

#### Thiobarbituric acid reactive substances (TBARS)

TBARS were determined by colorimetric method in plasma samples diluted with deionized water (1:5). Next, 1 mL of the diluted sample was transferred to glass tubes, 1 mL of trichloroacetic acid (TCA) 17.5% and 1 mL of thiobarbituric acid 0.6% (Sigma, St. Louis, MO, USA), pH 2 were added. Sample tubes were set in a water bath at 95°C for 20 minutes, and then cooled to room temperature. Next, 1 mL of TCA 70% was added and incubated for 20 minutes. Afterwards, incubated samples were centrifuged at 3,000 rpm for 20 minutes at 4°C and the absorbance read at 534nm on a microplate reader (Synergy HT, Biotek, Winooski, USA). TBARS concentration calculations were performed using the extinction coefficient, 1.56 × 10^5^ mol^-1^ cm^-1^, with plasma results expressed in nmol/ml.

#### Reduced/oxidized glutathione (GSH/GSSG)

Erythrocytes GSH/GSSG were measured by colorimetric assay (EnzyChrom GSH/GSSG Assay- EGTT-100) in a morning plasma sample collected after 8-12 hours fasting. This assay kit is designed to accurately measure total, reduced and oxidized glutathione in biological samples using an enzymatic method. Linear detection range 0.01-3 μM GSH equivalents with a detection limit of 10 nM GSH equivalents.

### Other laboratory tests

Lipid profile, which includes total cholesterol, LDL-cholesterol, HDL-cholesterol and triglycerides, were obtained by colorimetric assays of the 8-12 hour fasting plasma samples. Glycated hemoglobin (HbA1c) was obtained from HPLC (nv: 4.0–5.6%) (TOSOH G7 Luxembourg, Belgium). Ferritin was determined by electrochemiluminescence (Access, Beckman Coulter) and C-reactive protein (CRP) by immunoturbidimetric assays (Olympus AU 640). Albuminuria was measured in an isolated overnight urine aliquot by immunoturbidimetric assay (two positives in three samples) (normal value <20 μg/min).

### Statistical analysis

The sample size was calculated to get a power of 80% to detect a correlation ≥0.3 between variables studied, with α value of 5%.

Statistical evaluation was performed using Sigma Stat Version 3.5 (CA, USA). Numerical data were expressed as mean ± standard deviation. Parametric and nonparametric tests were used according to the distribution (normal or not, respectively) of the data studied.

For comparing two variables, when they presented an equal variance test, it was used the t-test. When equal variance test failed, it was used a Mann-Whitney test. To verify the relationship between quantitative variables was used the Spearman correlation coefficient. A p value < 0.05 was considered statistically significant.

## RESULTS

**Figure 1:**
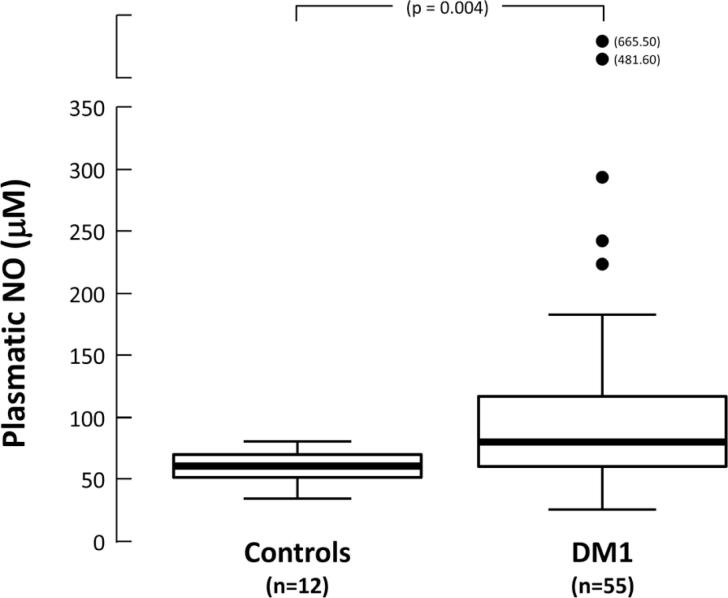
Distribution of plasmatic NO (μM) values found in the control and type 1 diabetes group (Bars = medium and SD of values).

Clinical and biochemical characteristics of controls and type 1 diabetes individuals studied are shown in Table 1. Age, BMI, STGV, LTGV, albuminuria, serum CRP and plasmatic NO were significantly different between controls and T1DM patients. Plasmatic NO was significantly higher in T1DM than controls (Figure 1), even after adjusted for age and BMI. There were no significant differences between the groups for gender distribution, serum lipid profile, ferritin, urinary 8-iso-PGF-2α, plasmatic TBARS and GSH/GSSG.

**Table 1:** The clinical and biochemical characteristics of controls and type 1 diabetes individuals studied

Characteristics	Controls	Type 1 diabetes	p value
N	22	76	
Age (years)	25.8 ± 3.9	23.6 ± 6.8	0.041
Gender (female/male)	15/7	35/41	0.090
Duration of T1DM (years)	-------------	13.0 ± 6.0	
BMI (kg/m²)	22.1 ± 2.8	23.8 ± 3.6	0.045
Insulin Dose (U/kg/day)	--------------	0.83 ± 0.28	
Cholesterol (mg/dL)	167.8 ± 29.9	164.4 ± 40.6	0.632
Triglycerides (mg/dL)	86.7 ± 43.8	103.3 ± 97.3	0.917
HDL cholesterol (mg/dL)	47.4 ± 13.8	44.5 ± 11.4	0.157
LDL cholesterol (mg/dL)	103.0 ± 27.2	100.3 ± 32.3	0.636
CRP (mg/L)	3.91 ± 2.72	4.99 ± 3.97	0.017
Ferritin (ng/mL)	104.9 ± 108.1	112.1 ± 100.9	0.571
Albuminuria (%)	0	17	0.042
STGV*	10.8 ± 1.7	74.5 ± 19.8	<0.001
LTGV**	5.4 ± 0.3 (35)	8.7 ± 1.6 (71)	<0.001
8-iso-PGF-2α (pg/mL)	1414.09 ± 557.14	941.22 ± 595.86	0.056
NO (μM)	63.8 ± 13.6	115 ± 104.1	0.004
TBARS (nmol/mL)	3.28 ± 1.62	3.35 ± 1.69	0.427
GSH/GSSG	4.73 ± 3.93	4.40 ± 3.14	0.857

* Standard Deviation (SD) over 3 consecutive days (CGMS).

** Mean glycated hemoglobin (HbA1c) of the last year.

In the T1DM group STGV showed positive correlations with serum total cholesterol (rS: 0.227; p:0.05) and triglycerides (rS: 0.241; p:0.03) while recording an inverse correlation with age (rS: −0.239, p: 0.037).

**Figure 2:**
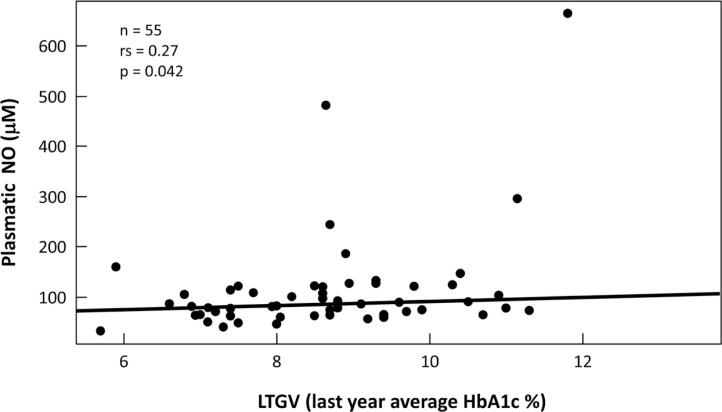
Correlation between plasmatic NO (μM) and LTGV (last year average HbA1c) (%) in T1DM.

LTGV (8.7± 1.6% or 71 ± 18 mmol/mol) showed a positive correlation with short-term glycemic variability (STGV) (rS: 0.361; p: 0.001), serum triglycerides (rS: 0.361; p: 0.001) and plasmatic NO (rS: 0.278; p: 0.042) (Figure 2).

**Figure 3:**
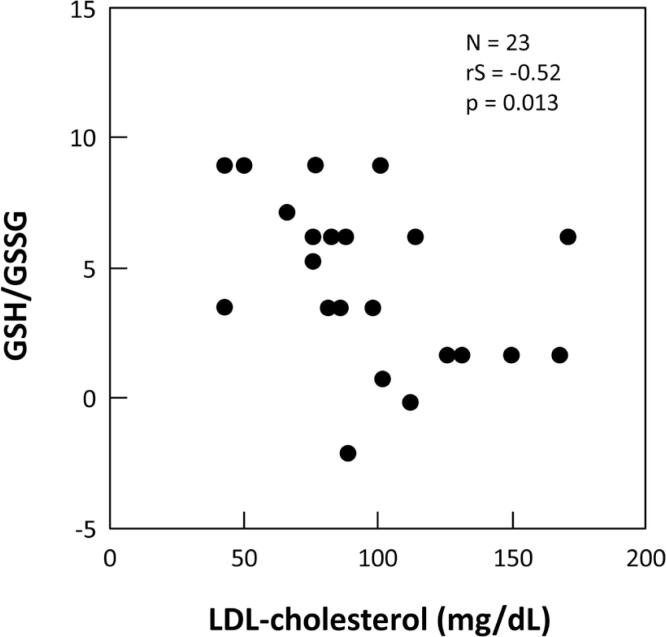
Correlation between GSH/GSSG and LDL-cholesterol in T1DM (mg/dL).

Both serum LDL-cholesterol and serum triglycerides were inversely correlated with GSH/GSSG (rS: −0.417; p: 0.047 and rS: −0.521; p: 0.013, respectively) (Figure 3) in T1DM.

**Figure 4:**
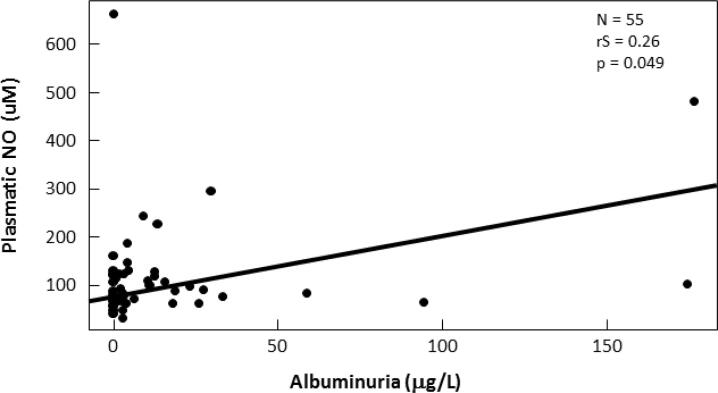
Correlation between plasmatic NO (μM) and albuminuria (mg/L) in T1DM.

**Figure 5:**
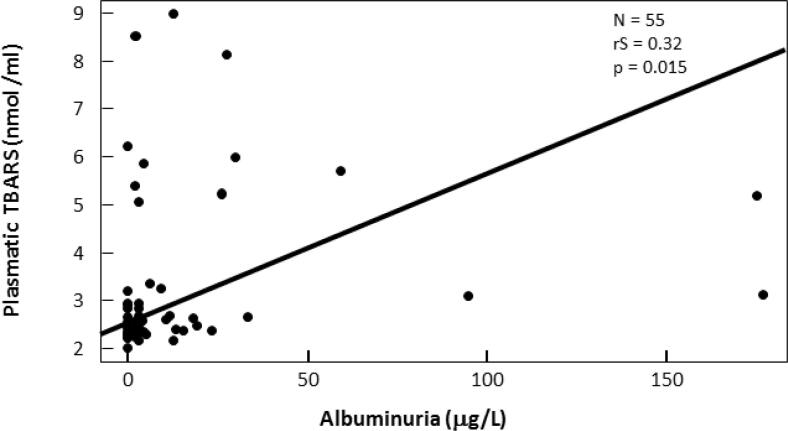
Correlation between plasmatic TBARS (nmol/ml) and albuminuria (mg/L) in T1DM.

Albuminuria was significantly positive correlated with age (rS: 0.340; p: 0.002), plasmatic NO (rS: 0.267; p 0.049) (Figure 4) and plasmatic TBARS (rS: 0.327; p: 0.015) (Figure 5) in T1DM.

## DISCUSSION

This study showed that plasmatic NO levels, an indicator of oxidative stress, were significantly higher in a subgroup of young adults with T1DM when compared to healthy age adjusted controls. However, the plasmatic TBARS and GSH/GSSG levels and urinary 8-iso-PGF2α excretion were similar between these two groups.

As expected, the glycemic variability was seven times higher in T1DM than in healthy controls. The glycemic excursions in these patients were positively correlated with plasmatic NO, while the lipid profile inverse correlated with GSH/GSSG and albuminuria positively correlated with plasmatic NO and TBARS.

The results regarding the relationship between glycemic variability and oxidative stress in T1DM are plenty and heterogeneous in the literature (15). In this present study, the only oxidative stress biomarker associated with long-term glycemic variability (LTGV) was the plasmatic NO levels. The others oxidative stress biomarkers studied didn't show any correlation with glycemic variability.

It has been described that T1DM have reduced NO bioavailability or diminished vascular response to NO, either because it is destroyed more rapidly by superoxide or due to decreased target enzyme responses. Therefore, basal NO synthesis must increase to maintain an equivalent level of basal NO-mediated dilatation in diabetic subjects and controls (16). This is also consistent with the observation that overall NO production is elevated rather than diminished in T1DM (17), what is in accordance with our data.

However, by measuring total value of plasma NO levels, it is not possible to distinguish if plasma NO levels were produced by eNOS or iNOS specifically. It has been demonstrated that high blood glucose levels inhibit endothelial production of NO (18). On the other hand, we know that hyperglycemia and oxidative stress increase iNOS activity, thereby accelerating NO synthesis (19). Other studies have found that hyperglycemia activates NF-πB, which induces the iNOS expression (20). Based on these data, it is reasonable to suggest that elevated NO levels in T1DM indicate increased iNOS expression and activity. Higher iNOS production of NO is in line with being a good and adaptable response to injury and inflammation. However, when NO expression is persistently up-regulated, NO is implicated in endothelial dysfunction, excessive vasodilation, extravasation and tissue injury (21). Some authors have suggested the importance of preventing excessive NO release mediated by iNOS, without suppression of eNOS in more favorable outcomes (20).

The other oxidative stress biomarker studied, 8-iso-PGF2α, in an 8-hour urine sample, showed great variability in both T1DM and controls and despite having a tendency, there was no significant difference between them. These results were like those found by other authors, who showed similar levels of this marker in controls comparing with, both T2DM treated with insulin and T1DM, but different from T2DM treated with oral medications (22). This suggests that insulin potentially exerts beneficial effects against the activation of oxidative stress dependent on sustained hyperglycemia and glycemic variability (23). So, a possible explanation for the similar levels of oxidative stress biomarkers, except plasmatic NO, in T1DM and controls in our study, is the potential inhibitory effect of the insulin therapy on oxidative stress (22). In addition, other factors that may influence the findings are the different methods for quantifying urinary 8-iso-PGF-2α (ELISA and mass spectrometry) (24) and the sample size.

Other authors found high levels of urinary 8-iso-PGF-2α in T1DM compared to healthy controls (9,15,25). Some studies have shown a correlation between this marker of oxidative stress and glycemic variability (9), while others (25,26) have not, suggesting the heterogeneity of the relationship between T1DM condition and oxidative stress biomarkers.

The results of many clinical and experimental studies have suggested that lipid peroxidation processes are activated during different stages of T1DM (27). However, in our study, plasmatic TBARS that can be considered as a lipid peroxidation index, were similar between the control group and T1DM patients with more than 5 years of disease, which was also observed by other authors (28).

Previous studies suggest that plasma lipid profile can also be affected by lipid peroxidation (27). Lower levels of antioxidant defenses can lead to higher levels of cholesterol, what could be seen in our study that found serum LDL-cholesterol and triglycerides inversely correlated with GSH/GSSG.

We found also that albuminuria was positively correlated with plasmatic NO and TBARS. This is in accordance with a study that had found elevated plasmatic TBARS levels in all albuminuric diabetic patients (29). Altered bioavailability of NO is a major contributor to endothelial dysfunction and as we known microalbuminuria may reflect a generalized vascular dysfunction (30). Taken together the plasmatic TBARS and NO levels could be also an index of initial kidney damage.

It is known that biomarkers of subclinical inflammatory response as serum CRP and ferritin have shown controversial results in T1DM patients (31,32). Mild increases of high sensitivity serum CRP are associated with a higher cardiovascular risk (33). In our study, CRP levels were significantly higher in the T1DM than in the control group, however this acute phase inflammatory protein did not correlate with oxidative stress parameters studied in this group of T1DM.

Ferritin concentration has been reported as a risk factor for the development of diabetes, impaired insulin sensitivity and cardiovascular disease (34), however in our study this parameter was similar in both controls and T1DM individuals and it did not correlate with oxidative stress parameters.

The current study has a few shortcomings. First, urinary nitric oxide was not measured, only the plasmatic sample. Also 8-iso PGF-2α was dosed in 8-hour urinary samples instead of 24-hour samples. Finally, this was a cross-sectional study, which precluded the possibility to follow up on patients and monitor the evolution of oxidative stress parameters.

The advantages of our study was the use of CGMS to calculate short term glycemic variability, which may be more important since seven point glucose measurements from SMBG, that was done in another studies, may not detect the full degree of glycemic variability.

In conclusion, young adult with T1DM had higher plasmatic NO than healthy young adults and it was, in this study, the single marker of oxidative stress correlated with glycemic variability. Plasmatic TBARS, GSH/GSSG and urinary 8-iso-PGF-2α didn't show correlation with this parameter. In addition, blood GSH/GSSG was positively correlated with lipid profile, and plasmatic NO and TBARS with albuminuria.

Therefore, the interrelationship between glycemic variability and oxidative stress markers in T1DM is heterogeneous. Lipid profile (one of cardiovascular risk component) and albuminuria (endothelial disfunction index) are associated with different oxidative stress markers during T1DM daily lives. Taken together this data collaborate to explain the different results found in the studies that searched for the correlation between glucose variability, oxidative stress and chronic diabetes consequences in T1DM. Further researches are needed, including prospective trials to better explore the long-term impact of the diabetic milieu.
